# A complex hepatitis B virus (X/C) recombinant is common in Long An county, Guangxi and may have originated in southern China

**DOI:** 10.1099/vir.0.026666-0

**Published:** 2011-02

**Authors:** Zhong-Liao Fang, Stéphane Hué, Caroline A. Sabin, Guo-Jian Li, Jin-Ye Yang, Qin-Yan Chen, Kong-Xiong Fang, Jian Huang, Xue-Yan Wang, Tim J. Harrison

**Affiliations:** 1 Division of Medicine, UCL Medical School, London, UK; 2 Guangxi Zhuang Autonomous Region Center for Disease Prevention and Control, Jin Zhou Road, Nanning, Guangxi, PR China; 3 Division of Infection, UCL Medical School, London, UK; 4 Division of Population Health, UCL Medical School, London, UK; 5 The Public Health Bureau of Guangxi Zhuang Autonomous Region, Tao Yuan Road, Nanning, Guangxi, PR China; 6 Long An Center for Disease Prevention and Control, ChengXi Road, Cheng Xiang Town, Long An, Guangxi, PR China

## Abstract

Recently, a complex (X/C) hepatitis B virus (HBV) recombinant, first reported in 2000, was proposed as a new genotype; although this was refuted immediately because the strains differ by less than 8 % in nucleotide distance from genotype C. Over 13.5 % (38/281) of HBV isolates from the Long An cohort in China were not assigned to a specific genotype, using current genotyping tools to analyse surface ORF sequences, and these have about 98 % similarity to the X/C recombinants. To determine whether this close identity extends to the full-length sequences and to investigate the evolutionary history of the Long An X/C recombinants, 17 complete genome sequences were determined. They are highly similar (96–99 %) to the Vietnamese strains and, although some reach or exceed 8 % nucleotide sequence difference from all known genotypes, they cluster together in the same clade, separating in a phylogenetic tree from the genotype C branch. Analysis of recombination reveals that all but one of the Long An isolates resembles the Vietnamese isolates in that they result from apparent recombination between genotype C and a parent of unknown genotype (X), which shows similarity in part to genotype G. The exception, isolate QL523, has a greater proportion of genotype C parent. Phylogeographic analysis reveals that these recombinants probably arose in southern China and spread later to Vietnam and Laos.

## INTRODUCTION

Hepatitis B virus (HBV), the prototype member of the family *Hepadnaviridae*, has a circular, partially dsDNA genome of about 3200 nt (Tiollais *et al.*, 1985). In 1988, Okamoto *et al.* classified HBV into four genotypes, A, B, C and D, by comparing 18 HBV isolates and suggested a sequence divergence over the entire genome greater than 8 % as the basis for defining a genotype (Okamoto *et al.*, 1988); this is also reflected by a divergence of more than 4 % in the surface ORF (Kramvis *et al.*, 2005). Subsequently, genotypes E–H were described (Arauz-Ruiz *et al.*, 2002; Naumann *et al.*, 1993; Norder *et al.*, 1994; Stuyver *et al.*, 2000). The HBV genotypes have distinct geographical distributions: genotype A is found predominantly in North-west Europe, North America, central and sub-Saharan Africa; genotypes B and C in South-east Asia, China and Japan; genotype D in the Mediterranean, the Middle East and India; genotype E in Africa; genotype F in native Americans, Polynesia, and Central and South America; genotype G in the USA and France; and genotype H in Central America (Norder *et al.*, 2004). Although the 8 % cut-off is entirely arbitrary, it has proved quite robust for classifying non-recombinant isolates of HBV into genotypes.

HBV recombination was first detected in sequential samples from infected individuals and among integrated sequences from cases of hepatocellular carcinoma (HCC) (Georgi-Geisberger *et al.*, 1992; Tran *et al.*, 1991) and was first detected using a phylogenetic approach in 1996 (Bollyky *et al.*, 1996). Recombination within and between genotypes has created complex patterns and altered the cladistic structure of HBV genotypes (Purdy *et al.*, 2008). For example, the B2 subgenotype proved to be a hybrid of genotypes B and C (Sugauchi *et al.*, 2002) and HBV genotypes E and G have also been shown to be recombinant, consisting partly of sequences derived from genotypes D and A, respectively (Bowyer & Sim, 2000; Fares & Holmes, 2002). There are indications from the analysis of HBV recombinants that at least one more genotype remains to be detected (Schaefer, 2007).

Recently, Tran *et al.* (2008) reported a complex (X/C) recombinant, which has a high similarity to the ‘aberrant strains’ among Vietnamese isolates reported by Hannoun *et al.* (2000) 8 years earlier. Phylogenetic analysis of the complete genome of these strains revealed a separate clade and it was suggested that this constitutes a new genotype, I (Tran *et al.*, 2008). This proposal was rejected by experts in HBV phylogeny because the genetic distance from genotype C is within the 8 % limit (Kurbanov *et al.*, 2008). Nonetheless, the proposal of a ninth genotype was supported by a report of isolates from Laos, which analysed a larger number of novel sequences and assigned them to two candidate subgenotypes, I1 and I2 (Olinger *et al.*, 2008). More recently, Phung *et al.* (2010) reported that the X/C recombinant is rare in Hanoi in the north of Vietnam.

The Long An cohort was established in 2004 to determine whether HBV basal core promoter mutations are a marker of a very high risk of developing HCC (Fang *et al.*, 2008a). Other viral factors may be associated with tumour development (Fang *et al.*, 2008b) and, in order to investigate the role of genotype, we determined the genotype of HBV from representative study subjects from the cohort, including individuals who developed HCC, using sequences from the surface ORF and the star program (Myers *et al.*, 2006) (http://www.vgb.ucl.ac.uk/starn.shtml) and the National Centre for Biotechnology (NCBI) HBV genotyping tool (http://www.ncbi.nlm.nih.gov/projects/genotyping/formpage.cgi). These analyses revealed that 71 and 14 % of the study subjects are infected with genotypes C and B, respectively, but 13.5 % (38/281) of the isolates were not assigned to a specific genotype. Comparison to sequences in GenBank using the blast program (Altschul *et al.*, 1997) revealed around 98 % identity to HBV recombinants described from Vietnam (Hannoun *et al.*, 2000; Tran *et al.*, 2008). The aim of this study was to determine whether this close identity to the Vietnamese isolates extends to the full-length sequences, to map accurately the points of recombination and to investigate the evolutionary history of the Long An X/C isolates.

## RESULTS

### Characterization of the complete genomes and various ORFs

Of the 38 isolates that could not be genotyped, 21 have deletions and 17 are complete, with a length of 3215 nt, as for genotypes B, C, F and H. These 17 full-length sequences share a mean genetic similarity of 97.8 % (range 96.2–99.0 %) with the Vietnamese strain VH24 (GenBank accession no. AB231908) (Hannoun *et al.*, 2000). When compared to representative full-length genomes of all known genotypes, the genetic distance between these Long An isolates and the reference sequences exceeds 8 % for all genotypes except genotype C, for which the genetic distance ranks between 6.6 % (isolate QL523) and 9.3 % (isolate XW233) and, in total, five of the sequences reach or exceed 8 % difference from genotype C over the entire genome (Supplementary Table S1, available in JGV Online).

**Fig. 1.**
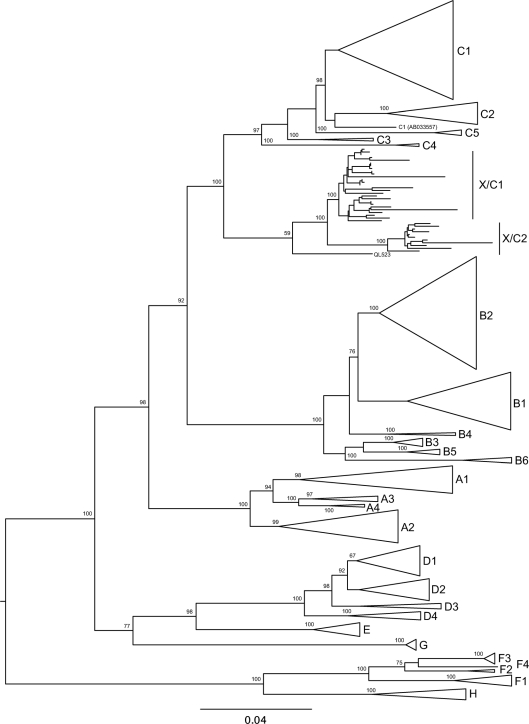
Maximum-likelihood phylogeny of 255 complete HBV genome sequences. Clusters of sequences of genotype other than X/C are represented by triangles labelled with the corresponding subgenotype. Numbers at the nodes represent the percentage of bootstrap resamples (1000 replicates) in which the node is supported (values >50 are shown). Bar, 0.04 nucleotide substitutions per site.

A phylogenetic tree constructed on the basis of the full-length genomes of our isolates is shown in Fig. 1. All of our strains and the recombinant strains from Vietnam and Laos form a cluster branching out from genotype C sequences, supported by a 100 % bootstrap value. This is with the notable exception of isolate QL523 which falls between the genotype C and recombinant clusters (Fig. 1). Again with the exception of isolate QL523, the recombinant sequences form two distinct subgroups [sequences from Laos (Olinger *et al.*, 2008) and the remainder], also supported by high bootstrap scores, suggesting diversification through two founder effects.

In the pre-S/S ORF, isolates QL523 and QQB36 encode residues L110, T126 and K160 in the major surface protein, which are characteristic of serological subtype *adr*, while other isolates have I110, T126 and K160, which are characteristic of subtype *adw* (Ohba *et al.*, 1995). No sequences encode a unique amino acid, as suggested previously (Tran *et al.*, 2008). Although seven conserved amino acid residues in the pre-S/S ORF, His^56^, Ala^60^, Asn^87^, Val^90^, Val^91^, Ile^136^ and Lys^198^, are unique compared with genotypes A, C and G (Tran *et al.*, 2008), they are shared with other genotypes. Calculation of phylogenetic distances reveals that, except for isolate QL523, the genetic difference between these surface ORF sequences and known genotypes exceeds 4 %. QL523 differs by only 3.5 % from genotype C, although the difference from other genotypes exceeds 4 %.

The P ORF is 2529 nt long in all strains, encoding a putative protein of 843 aa, as for genotypes B, C, F and H. S367 and S809 are unique amino acid residues compared with other genotypes. K267 also is revealed as a unique amino acid residue in all of the Long An sequences, except QL523. The complete C ORF is 636 nt long in all sequences and all sequences feature 1858. The X ORF is the same length as for other genotypes and no sequences predicted unique amino acids.

### Detection of recombination

**Fig. 2.**
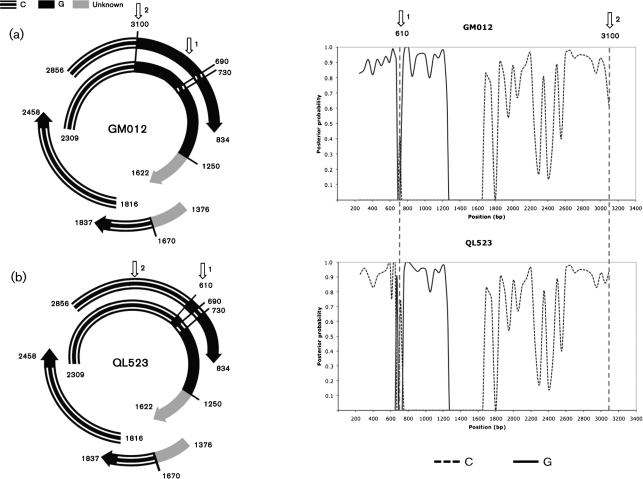
Detection of recombination breakpoints using a Bayesian MCMC sliding window approach. Isolates GM012 (a) and QL523 (b) were used as queries and compared to HBV sequences of genotypes A, B, C, D, E, F, G and H. The window size was set to 500 bp, with a step size of 50 bp. For reasons of clarity, only the most highly supported parental strain is shown in each window. Arrows labelled 1 and 2 indicate putative recombination breakpoints in GM012 and QL523.

Evidence of recombination was detected in all sequences by more than two programs (Martin *et al.*, 2005). Sliding window analyses identified three recombination breakpoints along the genome of 16/17 Long An isolates, dividing the genome into three distinct fragments (Fig. 2): (i) the region comprised between nucleotide positions 1670 and 3100 was more closely related to genotype C than to the other genotypes; (ii) the region spanning positions 1–1250 was closely related to genotype G; and (iii) the remaining part (positions 1250–1670) differed substantially from all known genotypes.

Isolate Ql523 had one different breakpoint and contained more of the putative genotype C parent (from position 1670 to 610) than the other 16 isolates. The remaining part was identical to that of the other Long An sequences. This suggests that isolate QL523 and the other isolates are the result of two independent recombination events involving the same parental strains.

### Geographical origin and dating of the X/C recombinant

**Fig. 3.**
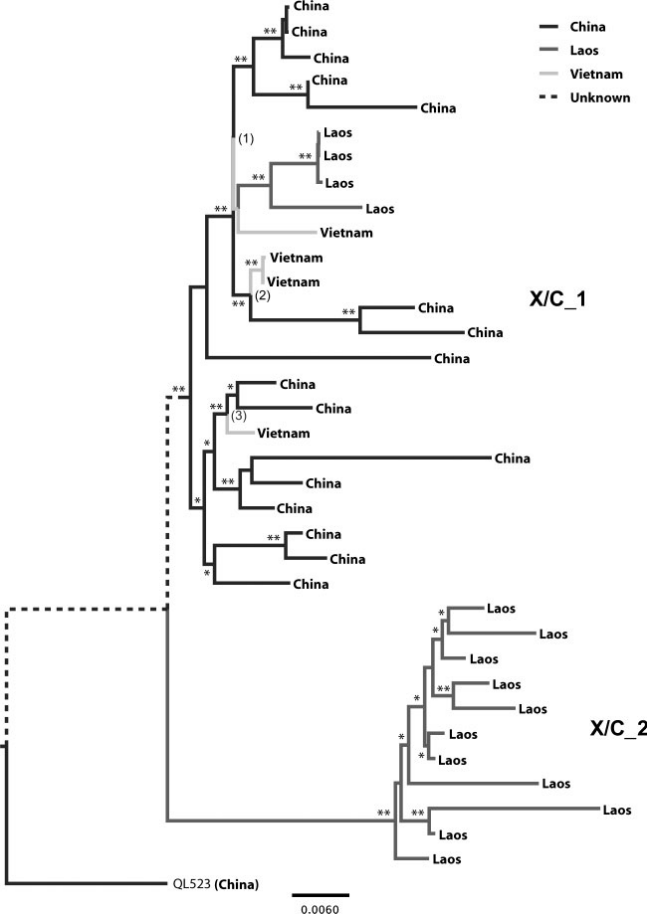
Bayesian ancestral reconstruction and migration patterns of the X/C recombinant, based on full-length sequences. Branches of the maximum clade credibility tree are shaded according to the most probable location of their descent node. Posterior location probabilities are indicated on the branches when >0.90 (*) or =1.00 (**). Numbers 1–3 indicate the three independent introductions of genotype X/C_1 into Vietnam at the corresponding nodes. Bar, 0.0060 nucleotide substitutions per site.

The spatial dispersion of the X/C recombinants was estimated by ancestral state reconstruction, using a Bayesian Markov chain Monte Carlo (MCMC) phylogenetic framework (Fig. 3). For reasons of clarity, the two distinct clades formed by the X/C isolates will be referred to as X/C_1 (24 sequences from Long An, Laos and Vietnam), and X/C_2 (11 sequences from Laos; see Fig. 3). Annotation of the phylogenetic nodes with their most probable origin suggests that the X/C_1 clade originated in southern China (Bayesian posterior probability of 1.00). The strain later was transferred to Vietnam on at least three different occasions (Bayesian posterior probability of 1.00). Clade X/C_1 was also introduced to Laos at least once. The origin of the X/C_2 subgroup could not be determined unambiguously by phylogeographic analysis, although the basal position of isolate QL523, from Long An, in the phylogenetic tree also suggests a Chinese origin.

**Fig. 4.**
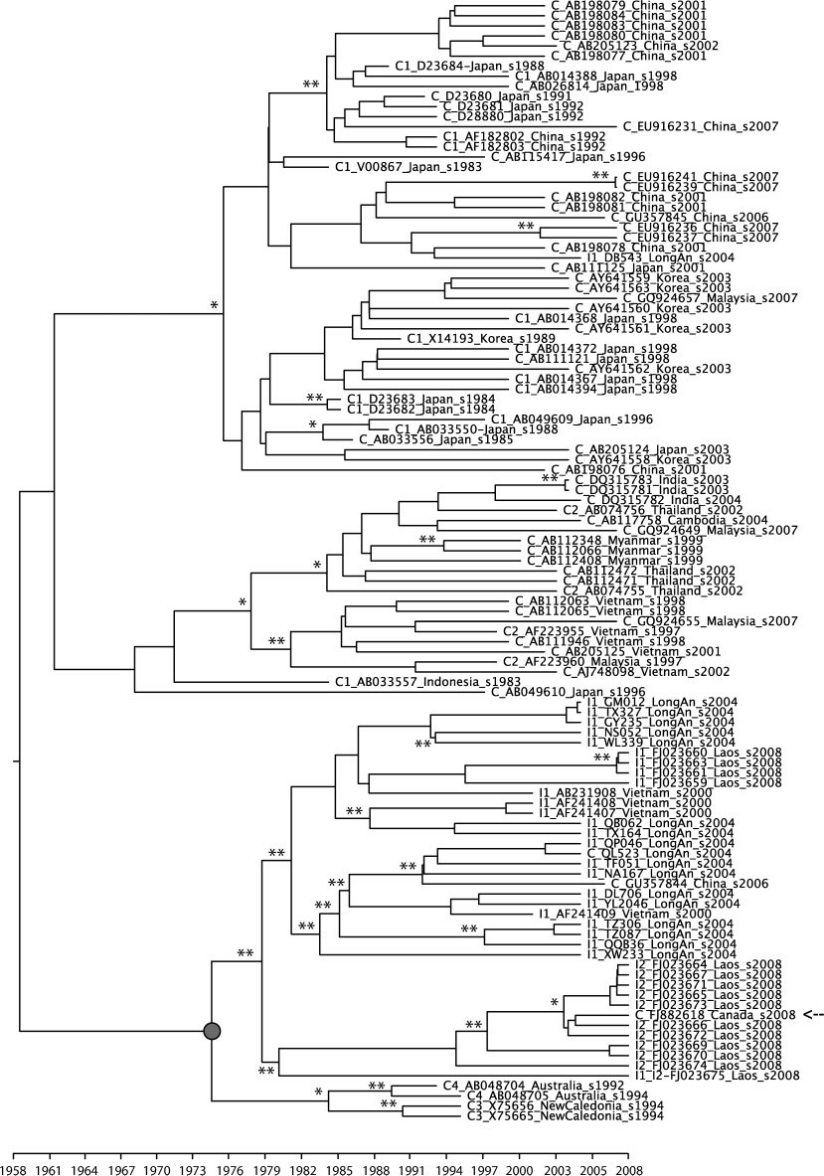
Dated Bayesian MCMC phylogenetic tree showing the relationship between the C-like portion of the X/C-recombinant (positions 1670–3100) and worldwide HBV genotype C isolates. Bayesian posterior probabilities are shown on the branches when >0.90 (*) or =1.00 (**). The most recent common ancestor of X/C, HBV genotype C3 and C4 is indicated by a filled circle. Branch lengths express years of divergence. A genotype C isolate (GenBank accession no. FJ882618), reported from Canada but of Vietnamese origin (Osiowy *et al.*, 2010) and closely related to X/C_2 sequences, is indicated by an arrow.

In order to investigate the probable origin of the C-like parental strain of the X/C recombinants, the corresponding regions were compared to HBV genotype C sequences of known geographical origin, using a Bayesian MCMC phylogeographic framework (Fig. 4). Surprisingly, the C-like fragment of the X/C recombinants is related more closely to subgenotype C3 and C4 sequences from New Caledonia and Australia, respectively (Bayesian posterior probability of 0.75), than to strains known to prevail in South-east Asia (i.e. subgenotypes C1 and C2). No close relative of the G-like fragment of the X/C isolates was found and its most likely geographical origin could not be determined unambiguously (data not shown).

### The prevalence of the recombinant in asymptomatic carriers and HCC patients

As stated in Methods, the 38 unassigned isolates are derived from 281 study subjects recruited as asymptomatic hepatitis B surface antigen carriers (Fang *et al.*, 2008a, 2009). Therefore, the prevalence of the recombinant is 13.5 % (38/281). Forty of the 281 subjects developed HCC during follow-up and nine of these 40 are infected by the recombinant, giving prevalences of 12.0 % (29/241) and 22.5 % (9/40) among the remaining asymptomatic carriers and HCC patients, respectively. This suggests that the prevalence of this unusual genotype is higher in HCC patients than in asymptomatic carriers, although the difference is not statistically significant (*χ*^2^=3.2, *P*>0.05).

## DISCUSSION

The major findings of this study are that the recombinant Long An sequences are highly similar (96–99 %) to the Vietnamese strains and some of them reach or exceed 8 % difference from all known genotypes, while the remainder differ by less than 8 % from genotype C. However, they all cluster in the same clade, separating from the genotype C branch of a phylogenetic tree based on complete genome sequences (Fig. 1). Furthermore, we have found that the prevalence of this X/C recombinant is quite high (13.5 %) in this region of southern China. These recombinants seem to have arisen in southern China and spread later to Vietnam and Laos; however, this conclusion is based on the best-fit origin among the small number of regions sampled and a wider geographical survey is required to determine definitively the region of origin.

This unusual recombinant was first reported in 2000 as aberrant strains (Hannoun *et al.*, 2000) and the mean genetic divergence from genotype C of <8 % over the entire genome dissuaded the authors from assigning these strains to a new genotype, although they suggested that further study of east Asian HBV sequences was required to establish the existence of a putative new genotype. In contrast, more recent reports suggested that these unusual recombinants do constitute a new genotype, I (Olinger *et al.*, 2008; Tran *et al.*, 2008), although these authors did not provide additional information or a new analytical approach. This proposal was refuted by experts on HBV phylogeny (Kurbanov *et al.*, 2008). Although we found some of our sequences reach or exceed 8 % in nucleotide divergence from all the established genotypes, they cluster in the same clade with those that exhibited less than 8 % nucleotide diversity from genotype C. The cut-off of 8 % nucleotide sequence difference, accepted for the assignment of new genotypes, clearly is not applicable to complex recombinant isolates.

In this analysis, recombination was detected using a suite of programs, implemented in the RDP2 package (Martin *et al.*, 2005), and mapped accurately using a sliding window analysis with construction of Bayesian trees. As noted previously, the recombinants seem to have arisen from genotype X and C parents, where the putative ‘genotype X’ itself may be the result of recombination between a genotype G-like isolate and an unknown parent. A surprising finding is that the genotype C parent of the X/C recombinants is more closely related to subgenotypes C3 and C4 than to C1. Of note, the cladistic separation of X/C_1 and X/C_2 (Fig. 3) is also evident in the analysis of the region derived from genotype C (Fig. 4). Critically, isolate QL523 has a different recombination breakpoint and a greater percentage of the genotype C parent than the other recombinant viruses, linking the recombinant clade more strongly to genotype C.

HBV gene exchange can occur between genotypes, within a genotype (Simmonds & Midgley, 2005) and between species (Magiorkinis *et al.*, 2005). About 87 % of the putative recombinants described up to now are B/C or A/D hybrids (Kramvis *et al.*, 2005). In this study, some recombinant sequences (minor parent) come from genotype C, which is quite common in southern China and, specifically, the Long An cohort. However, as noted above, phylogenetic analysis reveals that the C-like portion resembles subgenotypes C3 and C4 more closely than C1 and C2. The major parent (X) is unknown, although it is most similar to genotypes A, E and G and, if still extant, may itself constitute a novel, and perhaps recombinant, genotype. All samples in this study are from the Long An cohort (Fang *et al.*, 2008a), in southern Guangxi, a province of China which borders Vietnam. The distribution of this unusual genotype in other parts of Guangxi province and other regions in China is not known but it seems to have originated in southern China. We do not know when the putative recombination events may have occurred but the apparent absence of one parental strain (X) and the repeated introductions into Vietnam and Laos argue for an extensive period.

In this study, we found that the prevalence of this unusual genotype is higher in HCC than in asymptomatic carriers, although the difference is not statistically significant and further studies are required to clarify its relationship with HCC. HBV genotypes have been considered to impact on the pattern of mutations in the precore and core promoter regions and the natural course of infection, and may be associated with the severity of liver disease (including the development of HCC) and with response to treatment (Bottecchia *et al.*, 2008; Wang *et al.*, 2007). The double mutations A1762T/G1764A in the HBV basal core promoter have been confirmed as a causal factor of HCC (Fang *et al.*, 2008a), are more common in genotype B than C, and may be more common in the X/C recombinant than the pure genotype C (Z.-L. Fang and others, unpublished data). In addition, 21 of the 38 recombinant isolates identified in this study contained deletions, predominantly in the pre-S region, and pre-S deletions are common among HCC cases in the Long An cohort (Fang *et al.*, 2008b). Therefore, further studies are required to clarify the impact of this unusual recombinant on the pathological features of hepatitis B and its response to treatment.

## METHODS

### Study subjects and serological testing.

The Long An cohort has been described previously (Fang *et al.*, 2008a) and is made up principally of agricultural workers from rural Guangxi who do not have risk factors for exposure to HBV other than living in a region with a very high prevalence of infection among the general population. The 38 unassigned isolates were from 281 study subjects selected from the cohort, including individuals selected for analysis of viral loads (Fang *et al.*, 2009) and analysis of pre-S deletions (Fang *et al.*, 2008b). Genotypes of HBV from the isolates were determined using sequences from the surface ORF (Fang *et al.*, 2008b, 2009) except for 38 subjects whose genotypes were not assigned. Serological testing and HCC diagnosis were described in a previous report (Fang *et al.*, 2008a).

### Nested PCR for HBV DNA and nucleotide sequencing.

The full-length HBV genome of the 38 unassigned isolates was amplified using nested PCR. The first round amplification protocol and primers P1 and P2 have been described previously (Gunther *et al.*, 1995). The second round PCR was carried out on 5 μl of the first round products in a 50 μl reaction using primers MDN5R (nt 1774–1794, 5′-ATTTATGCCTACAGCCTCCT-3′) and BCPF (nt 1854–1875, 5′-ATGTCCTACTGTTCAAGCCTCC-3′), with 5 min hot start followed by 30 cycles of 94 °C for 30 s, 50 °C for 30 s and 72 °C for 4 min. Products from the second round were confirmed by agarose gel electrophoresis and then purified using GenElute PCR Clean-up kits (Sigma) according to the manufacturer's instructions. Cycle sequencing was carried out directly on both strands using 2 μl purified amplicon DNA and primers (Supplementary Table S2, available in JGV Online) and a BigDye Terminator V3.1 Cycle Sequencing kit (Applied Biosystems) according to the manufacturer's instructions. Sequences with deletions were sequenced on one strand only but the remainder were sequenced on both strands to derive robust data for comparison with the full sequences of the various genotypes.

### Phylogenetic analyses.

In order to determine the genotype of the Long An isolates, phylogenies were reconstructed on the basis of (i) the full-length sequences (3215 nt), and (ii) the S ORF (681 nt) of the viruses. The sequences were aligned to 198 HBV sequences of all known genotypes retrieved from GenBank, using the program clustal w2 (http://www.ebi.ac.uk), then manually corrected with the sequence editor BioEdit (http://www.mbio.ncsu.edu/BioEdit/bioedit.html). Maximum-likelihood trees were reconstructed under the General Time Reversible model of nucleotide substitution, with proportion of invariable sites and gamma-distributed rate heterogeneity (GTR+I+Γ), using the software paup* version 4.0b10 (Swofford, 1991). The robustness of the trees was assessed by bootstrap analyses, with 1000 replicates.

Pairwise genetic distances between the Long An and reference isolates were also calculated under the GTR+I+G model of evolution, using the program paup*, for both the full-length and the S gene sequences.

### Detection of recombination.

Potential recombinant sequences in the Long An isolates were detected using the programs RDP, Geneconv (Padidam *et al.*, 1999), MaxChi (Smith, 1992), Chimera (Posada & Crandall, 2001), Bootscan (Salminen *et al.*, 1995) and SisScan (Gibbs *et al.*, 2000) implemented in the RDP2 package (Martin *et al.*, 2005). General recombination settings for all programs were as follows: sequences were considered circular, the highest acceptable *P* value cut-off was set to 0.05, a Bonferroni correction was applied, phylogenetic evidence was required, breakpoints were polished, alignment consistency was checked for and overlapping signals were disentangled. SEQEN parametric simulations were used. Specific settings for each program were as follows: for RDP, no reference sequence was selected, and percentage of identity between recombinant sequences was set from 0 to 100. For Geneconv, sequence triplets were scanned, each indel was treated as a polymorphism and the g-scale was set to 1. For MaxChi, gaps were stripped and variable sites per window were set to 70. For Chimera, variable sites per window were set to 70. For Bootscan, the window size was set to 200 bp, step size to 20 bp and neighbour-joining trees used. The number of bootstrap replicates was 100, the cut-off percentage was 70 % and model options were set to the Kimura 2-parameter model (Kimura, 1980). For SisScan, window size was 200 bp, step size was 20 bp, gaps were stripped and the *P* value permutation number was 1000.

The recombinant breakpoints of suspected mosaic genomes were mapped using the program SlidingBayes (Paraskevis *et al.*, 2005). The window size was set to 500 bp, with a step size of 50 bp. Within each window, Bayesian trees were sampled every 1000th generation of 10 000 000 iterations and a maximum clade credibility tree (MCCT) was selected using the program FigTree (http://tree.bio.ed.ac.uk/software/figtree/). The trees were reconstructed under the GTR+G model of nucleotide substitution. The positions of identified breakpoints were confirmed by reducing the window step size to 20 bp in the regions flanking the breakpoints.

### Phylogeographic analyses.

Genotype X/C recombinants have been found in southern China, Laos and Vietnam. In order to investigate the spatial dispersion patterns of the viruses and attempt to identify the geographical origin of the recombinant form, phylogeographic analyses were conducted according to the Bayesian MCMC method developed by Lemey *et al.* (2009). Each X/C recombinant full-length sequence was assigned a geographical state corresponding to its country of sampling: Long An, southern China (*n*=17); Vietnam (*n*=4; Hannoun *et al.*, 2000); and Laos (*n*=15; Olinger *et al.*, 2008). Ancestral state reconstruction was then performed along the sequences’ phylogeny using the beast program version 1.5.2 (Drummond & Rambaut, 2007). Dated phylogenies were estimated using the General Time Reversible model of nucleotide substitution with gamma-distributed rate heterogeneity, a relaxed molecular clock and a Bayesian Skyline coalescent model. The Bayesian MCMC search was set to 5 000 000 iterations, with trees sampled every 1000th generation. An MCCT was selected from the sampled posterior distribution with the program TreeAnnotator version 1.5.2 (http://beast.bio.ed.ac.uk/), after discarding trees corresponding to a 10 % burnin. The MCCT was edited with the program FigTree version 1.1.2.

Bayesian MCMC phylogeographic analyses were also performed under the aforementioned conditions for the C-like (positions 1670–3100) and G-like fragment (positions 200–1250) of the X/C recombinant form only, in order to identify the most likely origin of the corresponding parental strains. The C-like fragment was compared to HBV genotype C1 sequences from China (GenBank accession nos AF182802, AF182803, AB198076–AB198084, AB205123, EU916231, EU916236, EU916237, EU916239, EU916241 and GU357845), Indonesia (AB033557), Japan (AB014367, AB014368, AB014372, AB014388, AB014394, AB026814, AB033550, AB033556, AB049609, AB111121, AB111125, AB115417, AB205124, D23680–D23684, D28880 and V00867), Malaysia (GQ924657) and South Korea (AY641558–AY641563 and X14193); genotype C2 sequences from Cambodia (AB117758), India (DQ315781–DQ315783), Japan (AB049610), Myanmar (AB112066, AB112348 and AB112408), Thailand (AB074755, AB074756, AB112471 and AB112472), Malaysia (AF223960, GQ924649 and GQ924655) and Vietnam (AB111946, AB112063, AB112065, AB205125, AF223955 and AJ748098); genotype C3 sequences from New Caledonia (X75656 and X75665); and genotype C4 from Australia (AB048704 and AB048705). The G-like fragment was compared to HBV genotype G sequences from Brazil (EF464097), France (EF634480 and EF634481), Germany (AF405706 and DQ207798), Italy (EF514346–EF514349), Japan (AP007264), Mexico (AF369533), the Netherlands (DQ403176 and GU565217) and the USA (AB056513 and AB064313); HBV C/G recombinants from Thailand (DQ078791 and FJ361772); HBV B/G recombinants from Taiwan (AB555499) and Japan (AB549213); and HBV G/A recombinants from Canada (EU833889 and EU833890).

### Nucleotide sequence accession numbers.

The nucleotide sequence data reported in this paper have been submitted to the GenBank/EMBL/DDBJ databases under accession numbers FR714490–FR714506.

## Supplementary Material

[Supplementary Tables]
