# Supplementary material for: A complex hepatitis B virus (X/C) recombinant is common in Long An county, Guangxi and may have originated in southern China
Source: J Gen Virol. 2011 Feb;92(Pt 2):402–11. doi: 10.1099/vir.0.026666-0 (PMC3081081; doi:10.1099/vir.0.026666-0)
Supplement: [Supplementary Tables] [file supp_92_2_402__index.html]

 A complex hepatitis B virus (X/C) recombinant is common in Long An county, Guangxi and may have originated in southern China -- Fang et al. 92 (2): 402 Data Supplement - Supplementary Tables -- Journal of General Virology

## 

### A complex hepatitis B virus (X/C) recombinant is common in Long An county, Guangxi and may have originated in southern China, by Z.-L. Fang, S. Hué, C. A. Sabine, G.-J. Li, J.-Y. Yang, Q.-Y. Chen, K.-X. Fang, J. Huang, X.-Y. Wang and T. J. Harrison

*Journal of General Virology* vol. **92**, part 2, pp. 402 - 411

**Supplementary Table S1.** Pairwise analysis of genetic distance – full genome (substitution/site)

**Supplementary Table S2.** Sequencing primers   
  
 [Single PDF file]  (58 KB)

  
  
