# Supplementary material for: A complex hepatitis B virus (X/C) recombinant is common in Long An county, Guangxi and may have originated in southern China
Source: J Gen Virol. 2011 Feb;92(Pt 2):402–11. doi: 10.1099/vir.0.026666-0 (PMC3081081; doi:10.1099/vir.0.026666-0)
Supplement: [Supplementary Tables] [file supp_92_2_402__1.pdf]

Supplementary Table S1. Pairwise analysis of genetic distance – full genome (substitution/site)

Isolate	Genotype															
	A		B		C		D		E		F		G		H	
	**Mean**	**SD**	**Mean**	**SD**	**Mean**	**SD**	**Mean**	**SD**	**Mean**	**SD**	**Mean**	**SD**	**Mean**	**SD**	**Mean**	**SDSD**
DL706	0.086	0.006	0.095	0.005	0.079	0.004	0.110	0.003	0.107	0.001	0.150	0.003	0.117	0.001	0.152	0.004
GM012	0.087	0.006	0.097	0.005	0.074	0.004	0.109	0.003	0.110	0.001	0.151	0.003	0.118	0.001	0.111	0.004
GY235	0.087	0.006	0.097	0.005	0.073	0.004	0.108	0.003	0.109	0.001	0.152	0.003	0.117	0.001	0.152	0.004
NA167	0.086	0.005	0.096	0.005	0.079	0.004	0.105	0.003	0.105	0.001	0.150	0.003	0.116	0.001	0.150	0.004
NS052	0.092	0.006	0.103	0.004	0.080	0.004	0.112	0.002	0.115	0.001	0.155	0.002	0.123	0.001	0.157	0.004
QB062	0.093	0.006	0.101	0.005	0.083	0.004	0.115	0.003	0.115	0.001	0.158	0.002	0.123	0.001	0.161	0.004
QL523	0.094	0.004	0.099	0.005	0.066	0.006	0.111	0.003	0.110	0.001	0.154	0.002	0.125	0.001	0.154	0.004
QP046	0.087	0.005	0.099	0.005	0.079	0.004	0.108	0.003	0.108	0.001	0.152	0.003	0.119	0.001	0.152	0.004
QQB36	0.089	0.006	0.098	0.005	0.078	0.004	0.109	0.003	0.111	0.001	0.153	0.003	0.117	0.001	0.152	0.004
TF051	0.099	0.005	0.109	0.005	0.092	0.004	0.120	0.003	0.120	0.001	0.165	0.003	0.130	0.001	0.164	0.003
TX164	0.089	0.005	0.097	0.005	0.077	0.004	0.111	0.003	0.111	0.001	0.153	0.002	0.120	0.001	0.154	0.004
TX327	0.089	0.005	0.098	0.005	0.075	0.004	0.110	0.003	0.110	0.001	0.153	0.003	0.119	0.001	0.152	0.004
TZ087	0.087	0.005	0.097	0.005	0.078	0.004	0.110	0.003	0.110	0.001	0.151	0.003	0.117	0.001	0.152	0.004
TZ306	0.089	0.006	0.099	0.004	0.081	0.004	0.114	0.002	0.112	0.001	0.154	0.002	0.120	0.001	0.154	0.004

Fang, Z.-L., Hué, S., Sabine, C. A., Li, G.-J., Yang, J.-Y., Chen, Q.-Y., Fang, K.-X., Huang, J., Wang, X.-Y. and Harrison, T. J. (2011).
A complex hepatitis B virus (X/C) recombinant is common in Long An county, Guangxi and may have originated in southern China. *J Gen Virol* **92**, 402–411.

WL339	0.088	0.006	0.098	0.005	0.074	0.004	0.108	0.003	0.110	0.001	0.152	0.002	0.119	0.001	0.153	0.004
XW233	0.102	0.006	0.111	0.005	0.093	0.004	0.123	0.003	0.126	0.001	0.170	0.004	0.129	0.001	0.169	0.004
YL2046	0.088	0.005	0.099	0.005	0.079	0.004	0.108	0.003	0.107	0.001	0.152	0.003	0.118	0.001	0.152	0.004

Fang, Z.-L., Hué, S., Sabine, C. A., Li, G.-J., Yang, J.-Y., Chen, Q.-Y., Fang, K.-X., Huang, J., Wang, X.-Y. and Harrison, T. J. (2011).
A complex hepatitis B virus (X/C) recombinant is common in Long An county, Guangxi and may have originated in southern China. *J Gen Virol* **92**, 402–411.

Supplementary Table S2. Sequencing primers

**Forward**		**Reverse**	
LSB1	nt 2809–2829, 5′-TTGTGGGTCACCATATTCTT-3′	MDC1	nt 2304–2324, 5′-TTGATAAGATAGGGGCATTTG-3′
ds	nt 147–166, 5′-TACCGAAAATGGAGAACACA-3′	POLSEQ5	nt 2979–2999, 5′-GGGTCCTTGTTGGGGTTGAAG-3′
MD14	nt 418–433, 5′-GCGCTGCAGCTATGCCTCATCTTC-3′	PSISEQ2	nt 65–84, 5′-GCTGTTCCGGAATTGGAGCC-3′
POLSEQ6	nt 1089–1109, 5′-TTTCACTTTCTCGCCAACTTA-3′	ADELN	nt 432–453, 5′-TAGTCCAGAAGAACCAACAAG-3′
BCPF	nt 1854–1875, 5′-ATGTCCTACTGTTCAAGCCTCC-3′	POLSEQ2	nt1168–1188, 5′-AGCAAACACTTGGCATAGGC-3′
POLSEQ4	nt 2305–2323, 5′-AAATGCCCCTATCTTATCAA-3′	MDN5R	nt 1774–1794, 5′-ATTTATGCCTACAGCCTCCT-3′

Fang, Z.-L., Hué, S., Sabine, C. A., Li, G.-J., Yang, J.-Y., Chen, Q.-Y., Fang, K.-X., Huang, J., Wang, X.-Y. and Harrison, T. J. (2011). A complex hepatitis B virus (X/C) recombinant is common in Long An county, Guangxi and may have originated in southern China. *J Gen Virol* **92**, 402–411.
